# Modulation of miR-146b Expression during Aging and the Impact of Physical Activity on Its Expression and Chondrogenic Progenitors

**DOI:** 10.3390/ijms241713163

**Published:** 2023-08-24

**Authors:** Luca Dalle Carbonare, Arianna Minoia, Michele Braggio, Jessica Bertacco, Francesca Cristiana Piritore, Sharazed Zouari, Anna Vareschi, Rossella Elia, Ermes Vedovi, Cristina Scumà, Matilde Carlucci, Lekhana Bhandary, Monica Mottes, Maria Grazia Romanelli, Maria Teresa Valenti

**Affiliations:** 1 Department of Engineering for Innovative Medicine, University of Verona, 37100 Verona, Italy; luca.dallecarbonare@univr.it (L.D.C.); arianna.minoia@univr.it (A.M.); michele.braggio@univr.it (M.B.); sharazed.zouari@univr.it (S.Z.); anna.vareschi@univr.it (A.V.); rossella.elia@univr.it (R.E.); 2 Department of Neurosciences, Biomedicine and Movement Sciences, University of Verona, 37100 Verona, Italy; jessica.bertacco@univr.it (J.B.); francescacristiana.piritore@univr.it (F.C.P.); monica.mottes@univr.it (M.M.); mariagrazia.romanelli@univr.it (M.G.R.); 3 Recovery and Functional Rehabilitation, Integrated University Hospital of Verona, 37100 Verona, Italy; ermes.vedovi@aovr.veneto.it (E.V.); cristina.scuma@aovr.veneto.it (C.S.); 4 Health Directorate, Integrated University Hospital of Verona, 37100 Verona, Italy; matilde.carlucci@aovr.veneto.it; 5 Flaskworks, LLC., Boston, MA 02118, USA; lekhana.bhandary@gmail.com

**Keywords:** miR-146b-5p, differentiation, circulating microRNAs, aging

## Abstract

The finding of molecules associated with aging is important for the prevention of chronic degenerative diseases and for longevity strategies. MicroRNAs (miRNAs) are post-transcriptional regulators involved in many biological processes and miR-146b-5p has been shown to be involved in different degenerative diseases. However, miR-146b-5p modulation has not been evaluated in mesenchymal stem cells (MSCs) commitment or during aging. Therefore, the modulation of miR-146b-5p in the commitment and differentiation of mesenchymal cells as well as during maturation and aging in zebrafish model were analyzed. In addition, circulating miR-146b-5p was evaluated in human subjects at different age ranges. Thus, the role of physical activity in the modulation of miR-146b-5p was also investigated. To achieve these aims, RT (real-time)-PCR, Western blot, cell transfections, and three-dimensional (3D) culture techniques were applied. Our findings show that miR-146b-5p expression drives MSCs to adipogenic differentiation and increases during zebrafish maturation and aging. In addition, miR-146b-5p expression is higher in females compared to males and it is associated with the aging in humans. Interestingly, we also observed that the physical activity of walking downregulates circulating miR-146b-5p levels in human females and increases the number of chondroprogenitors. In conclusion, miR-146b-5p can be considered an age-related marker and can represent a useful marker for identifying strategies, such as physical activity, aimed at counteracting the degenerative processes of aging.

## 1. Introduction

The study of the molecular regulation involved in aging may allow the identification of strategies for the “healthy aging” [1,2,3]. Cellular senescence plays an important role in inducing alterations involving the skeleton with repercussions on the quality of life. MicroRNAs (miRNAs) are post-transcriptional regulators involved in many biological processes, including cell growth, death, development, and differentiation.

Among miRNAs associated with degenerative diseases, miR-146b-5p is involved in the progression of colorectal cancer [4], in the aggressiveness and progression of thyroid cancer [5,6], and in acute ischemic stroke [7]. It has also been demonstrated that miR-146b regulates the adipogenic proliferation and differentiation in human visceral preadipocytes [8] and participates in the inflammatory process [9]. In addition, it has been reported that miR-146b-5p has a sex-specific involvement in renal and cardiac pathology [10]. Although miR-146b has been explored in many physio-pathological conditions, its expression has not been investigated in detail during aging. 

Recently, the *Danio rerio* (zebrafish) was proposed as a model for the study of vertebrate aging. In fact, the zebrafish can live on average for about 3 years (but also over 5 years in a controlled environment) and shows gradual aging that reflects what has been observed in humans [11]. The elderly zebrafish are characterized by a typical spinal curvature in addition to the reduction of regenerative capacity and degenerative processes [12]. The zebrafish is mainly used as an experimental model to understand the molecular mechanisms related to stem cells differentiation, their application in regenerative medicine, and as a model for drug discovery [13]. Therefore, it is relevant to investigate the modulation of miRNAs in developmental biology as well during the aging of this vertebrate model. 

Based on our previous studies, in which we have demonstrated the presence of miR-146b-5p in caudal fins of zebrafish that contain mesenchymal cells [14,15], we hypothesize that miR-146b-5p may have a role in osteochondrogenic lineage. Thus, with the aim of exploring the modulation of miR-146b-5p during development and aging, we used the zebrafish model in the larval as well as in the adult stages, including the aging stage, using the caudal fins. 

Additionally, in order to identify an aging marker in humans, we also explored the modulation of circulating miR-146b-5p in human subjects. 

Importantly, sedentary lifestyle in modern society produces various alterations at the basis of chronic degenerative diseases and physical activity represents a useful tool for human resilience, counteracting the risk of chronic diseases. Therefore, we evaluated the effects of a walking program in circulating miR-146b-5p levels as well its effects on osteogenic or chondrogenic differentiation. 

## 2. Results

### 2.1. MiR-146b Modulation during Differentiation of MSCs

**Figure 1:**
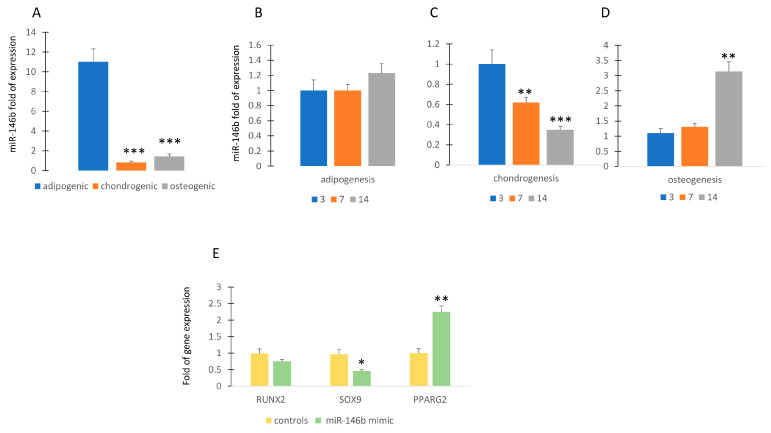
MiR-146b expression during differentiation. MiR-146b expression is higher in MSCs committed to adipogenic lineage compared to chondrogenic or osteogenic lineage (miR-146b expression in chondrogenic or osteogenic groups compared to adipogenic group) (**A**). MiR-146b expression is poorly modulated during adipogenesis (miR-146b expression at 7 or 14 days of differentiation compared to 3 days of differentiation) (**B**) and highly modulated during chondrogenic (**C**) or osteogenic (**D**) differentiation. Forced expression of miR-146b induced the upregulation of *PPARG2* and the downregulation of *SOX9* (**E**). Data are shown as mean  ±  standard deviation (SD); * *p* < 0.05; ** *p* < 0.01; *** *p* < 0.005. For each experiment, six independent analyses were performed. In figure (**B**–**D**), miR-146b expression at 7 or 14 days of differentiation is compared to that at 3 days of differentiation.

To investigate the role of miR-146b during differentiation, we analyzed its expression during the commitment and the differentiation of MSCs. As shown in Figure 1A, miR-146b is upregulated in MSCs committed to adipogenic lineage, whereas its expression is lower during osteogenic or chondrogenic commitment. 

MiR-146b continues to be expressed at similar levels during adipogenic differentiation (Figure 1B). In contrast, the expression miR-146b was reduced or increased during chondrogenic or osteogenic differentiation, respectively (Figure 1C,D). Forced expression of miR-146b obtained by transfecting MSCs with miR-146b mimic induced the upregulation of the adipogenic transcription factor *PPARG2*, while the expression of the chondrogenic transcription factor *SOX9* was reduced compared to controls (Figure 1E).

### 2.2. MiR-146b Is Associated with SESTRIN Expression

**Figure 2:**
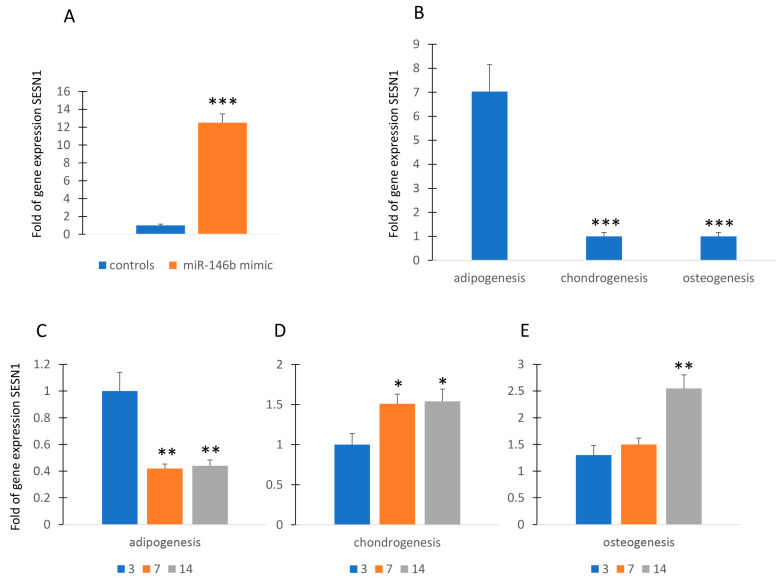
MiR-146b and *SESTRIN*. Forced expression of miR-146b increased *SESN1* gene expression in MSCs (**A**). *SESN1* is upregulated in adipogenic committed MSCs (**B**). *SESN1* expression was downregulated during adipogenesis (**C**) and upregulated during chondrogenesis and osteogenesis (**D**,**E**). Data are shown as mean ± standard deviation (SD); * *p* < 0.05; ** *p* < 0.01; *** *p* < 0.005. For each experiment, six independent analyses were performed.

Since cell differentiation, particularly adipogenic differentiation, is associated with physiological levels of stress [16,17], we wanted to investigate a possible relationship between miR-146b and *SESTRIN* (*SESN*), a marker of stress response. As shown in Figure 2A, forced expression of miR-146b in MSCs induced the increase in gene expression of SESN1 in MSCs. 

Therefore, we evaluated the expression of SENS1 during the commitment to adipogenic, chondrogenic, and osteogenic lineage. As shown in Figure 2B, commitment to adipogenesis induced the upregulation of *SESN1* while this gene was underexpressed in MSCs committed to chondrogenic or osteogenic lineage. However, the *SESN1* was downregulated during adipogenesis (Figure 2C). In contrast, *SESN1* expression was upregulated during chondrogenesis and osteogenesis (Figure 2D,E).

### 2.3. MiR-146b Increases during Zebrafish Maturation and Aging 

**Figure 3:**
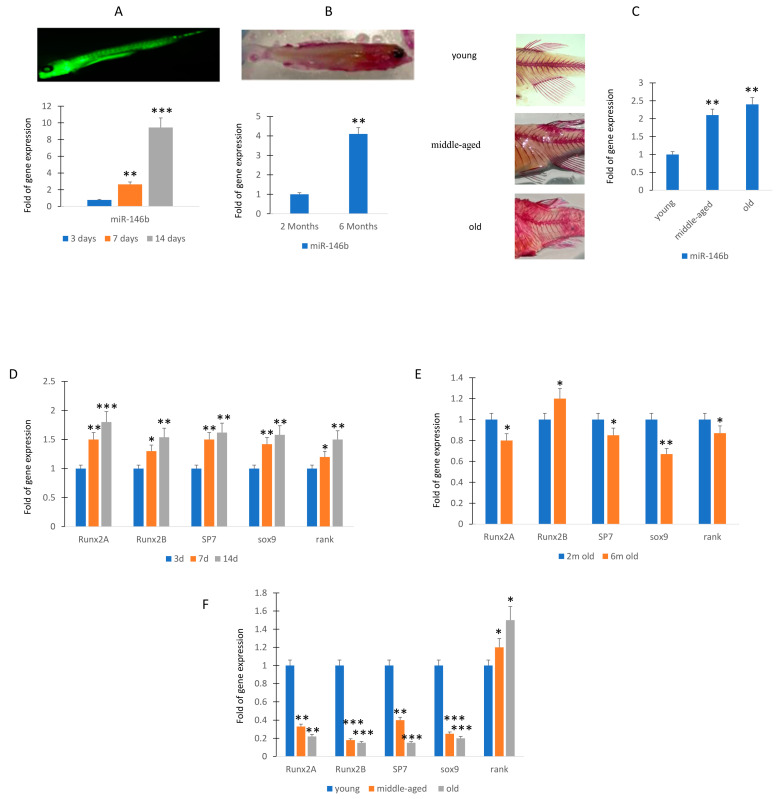
MiR-146b expression during zebrafish maturation. RT (real-time)-PCR analyses. MiR-146b expression after 3 (3d; sample *n* = 30), 7 (7d; sample *n* = 30), and 14 (14d; sample *n* = 30) days post fecundation (**A**). Expression levels of miR-146b at 2 (sample *n* = 30) and 6 months (sample *n* = 30) (**B**) and in young (sample *n* = 30), middle-aged (sample *n* = 30), and old zebrafish (sample *n* = 30) (**C**). Osteogenic (*runxa*, *runxb*, *sp7*), chondrogenic (*sox9*), and the receptor for RANK-Ligand gene expression increased during the maturation of larvae (**D**). *Runx2a* and *sox9* gene expression was downregulated in 6 months compared to 2 months old zebrafish (**E**). Osteogenic or chondrogenic differentiation associated genes lowered in caudal fin during the aging (**E**). Rank gene expression, involved in osteoclast activation, was higher in the caudal fin of middle-aged and old zebrafish (**F**). Data are shown as mean ± standard deviation (SD); * *p* < 0.05; ** *p* < 0.01; *** *p* < 0.005. (**A**): Calcein staining of zebrafish at 14 days post fertilization. (**B**): Alizarin staining of zebrafish at 6 months of age. In particular, at 14dpf the miR-146b increased more than 8-fold compared to 3 dpf (**A**). Therefore, we evaluated the miR-146b expression in adult caudal fin of zebrafish, a model system for the evaluation of molecular mechanisms involved in regeneration [18]. In particular, miR-146b expression was evaluated in young (*n* = 30; 10–12 months), middle-aged (*n* = 30; 20–24 months), and old (*n* = 30; 30–36 months) zebrafish samples.

To evaluate the expression of miR-146b in zebrafish, an animal model suitable for development and aging studies, we first analyzed the expression levels in larvae of zebrafish at 3, 7, and 14 days post fecundation (dpf). As shown in Figure 3A, the levels of expression increased during the maturation. 

In the younger adult phase, miR-146b continued to increase and we observed that the expression of miR-146b was more than 3-fold higher in 6-month-old zebrafish compared to the 2-month-old (Figure 3B). Then, we evaluated morphological changes of zebrafish skeleton occurring in the aging. In particular, we analyzed the spine modifications in young and 3-year-old zebrafish. As shown in Figure 3C, we observed an increase in curvature in middle-aged and this feature was more pronounced in old zebrafish. Therefore, we analyzed the RNA expression. The expression of miR-146b increased significantly in middle-aged and older specimens (Figure 3C),

To evaluate a relationship between miR-146b and differentiation markers in osteochondrogenic progenitors, we analyzed osteogenic and chondrogenic gene expression in larvae or caudal fin zebrafish. In the larvae of zebrafish at 3, 7, and 14 days dpf, the increased expression of genes involved in osteogenic differentiation (*runxa*, *runxb*, *sp7*) and chondrogenic differentiation (*sox9*) or bone remodeling (receptor for RANK-Ligand, *rank*) mirror the miR-146b expression modulation, being higher at 14 days (Figure 3D).

In contrast, we observed in 6-month-old zebrafish the downregulation of *runx2a* and *sox 9* gene expression and a negative trend of expression in the other investigated genes (sp7 and rank), whereas *runx2b* increased even if in a manner not statistically significant (Figure 3E). In addition, the expression of genes associated with osteogenic or chondrogenic differentiation, *runx2a*, *runx2b*, *sp7*, and *sox9* lowered in fin during aging (Figure 3F). However, the expression of rank, involved in osteoclast activation, increased during aging (Figure 3F). 

### 2.4. MiR-146b Expression Is Higher in Female and Increases during the Aging 

**Figure 4:**
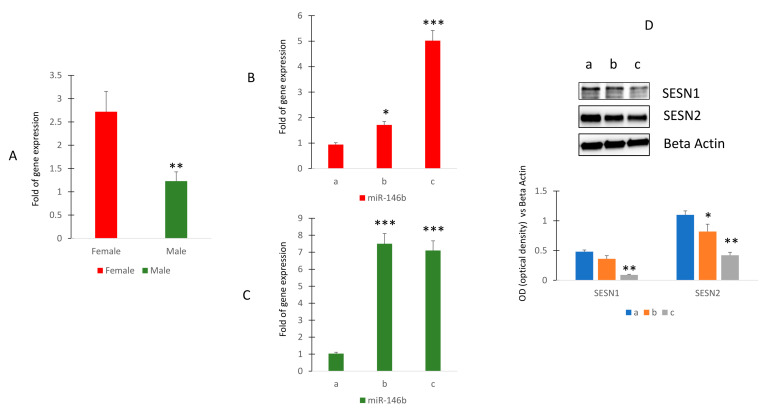
Circulating miR-146b expression in healthy human subjects. RT (real-time)-PCR in circulating miRNAs isolated from female (sample *n* = 12) and male (sample *n* = 17) (**A**) or in female (**B**) or in male (**C**) of different ages. Sestrin 1 and Sestrin 2 protein levels in MSCs cultured in presence of sera collected in women of different ages (**D**). (Median age: women 41.4 ± 7.2 ((group a (*n* = 4) 27.8 ± 1.9; group b (*n* = 4) 41.2 ± 1.9; group c (*n* = 4) 52.8 ± 0.8)); men 41.7 ± 9.7 ((group a (*n* = 5) 28.4 ± 1.1; group b (*n* = 6) 42.5 ± 0.8; group c (*n* = 6) 52.8 ± 0.63)). The original blots are presented in Supplementary S1. * *p* < 0.05; ** *p* < 0.01; *** *p* < 0.005.

To investigate the modulation of miR-146b expression in human subjects, we first evaluated any differences in the expression of this miR due to gender. Our results showed that circulating miR-146b expression was higher in female than in male human subjects (Figure 4A). 

Therefore, we evaluated the modulation of miR-146b during aging. Thus, we selected female subjects within the age range of 27 to 53 years (group a: 27.8 ± 1.9; group b: 41.2 ± 1.9; group c: 52.8 ± 0.8). Additionally, male participants within the similar age range were also included (group a: 28.4 ± 1.1; group b: 42.5 ± 0.8; group c: 52.8 ± 0.63). The findings revealed that among these individuals, circulating levels of miR-146b exhibited a noticeable rise with advancing age in females (Figure 4B), whereas in males, this increase became evident around the age of 42 and subsequently remained relatively constant (C). However, when we evaluated Sestrin levels in cultured mesenchymal cells in the presence of serum obtained from women of different ages, we observed reduced levels of Sestrin 1 and Sestrin 2 in serum-stimulated cells from older subjects (Figure 4C and Supplementary S1). 

### 2.5. Walking Program Effects on Circulating miR-146b and on Differentiation and Aging of MSCs

**Figure 5:**
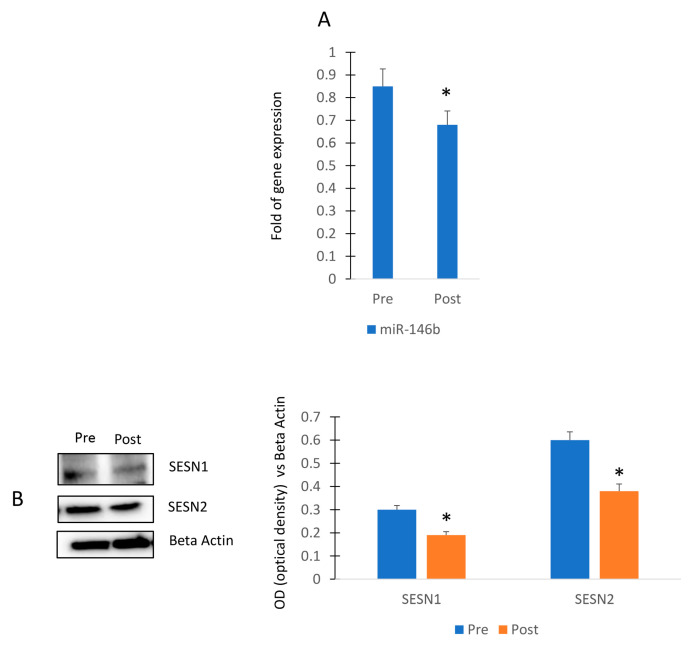
Walking program performed by female participants and miR-146b modulation. RT (real-time)-PCR in circulating miRNAs (Pre) and after (Post) the exercise program (**A**). Sestrin 1 and Sestrin 2 protein levels in MSCs cultured in presence of sera collected before (Pre) and after (Post) the exercise program (**B**). The original blots are presented in Supplementary S1. Data are shown as mean ± standard deviation (SD); * *p* < 0.05.

To evaluate the impact of physical activity in miR-146b modulation, we collected sera from female participants of a walking program (Figure 5A). In particular, the exercise program consisted of 3 walking sessions per week for a total of 4 weeks and sera were collected before (Pre) and after (Post) the 4-week session. As shown in Figure 5A, we observed that the walking program reduced the expression of circulating miR-146b. In addition, we observed reduced levels of both Sestrin 1 and Sestrin 2 in MSCs cultured in presence of sera collected after the exercise program (Figure 5B).

**Figure 6:**
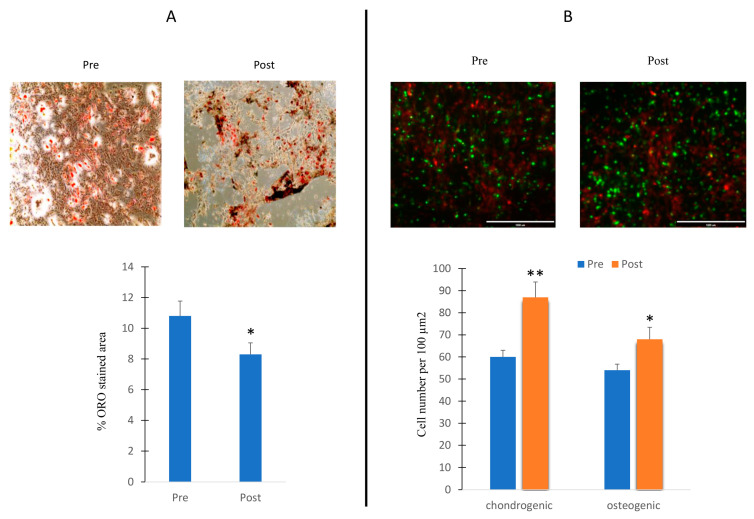
Effects of sera in differentiating cells. MSCs cultured during adipogenesis in presence of sera collected before (Pre) or after (Post) the exercise program. ORO-stained area in cells in presence of Pre and Post sera stimulation (**A**); 3D cultures of chondroblasts (green) and osteoblasts (red) in presence of Pre and Post sera stimulation (**B**). The increased chondroblast intensity observed in the post-treatment phase may potentially be attributed to cell overlap. Data are shown as mean  ±  standard deviation (SD); * *p* < 0.05; ** *p* < 0.01;. Magnification 10× (**A**) and 20× (**B**).

Thus, we cultured MSCs in adipogenic medium and in the presence of sera collected before and after the exercise program. Oil Red O (ORO) staining was performed in order to evaluate the number of oil droplet positive cells. As shown in Figure 6A, an increased ORO area was observed in cells under serum collected after the exercise program stimulation.

We also cultured osteogenic and chondrogenic cells in a 3D model in the presence of sera collected before and after the exercise program. An increase in the number of progenitor cells, especially in the chondrogenic lineage, has been observed in the presence of serum collected after the exercise program (Figure 6B). 

Since the p53/p21 axis has been associated with aging [19], we also evaluated the expression of p21and p53 in osteogenic or chondroblastic progenitors in presence of sera collected before and after the activity. The levels of MMP13, a metalloproteinase involved in cartilage degradation and associated with osteoarthritis [20], were evaluated as well in chondroblastic progenitors in the presence of sera collected before and after the activity.

**Figure 7:**
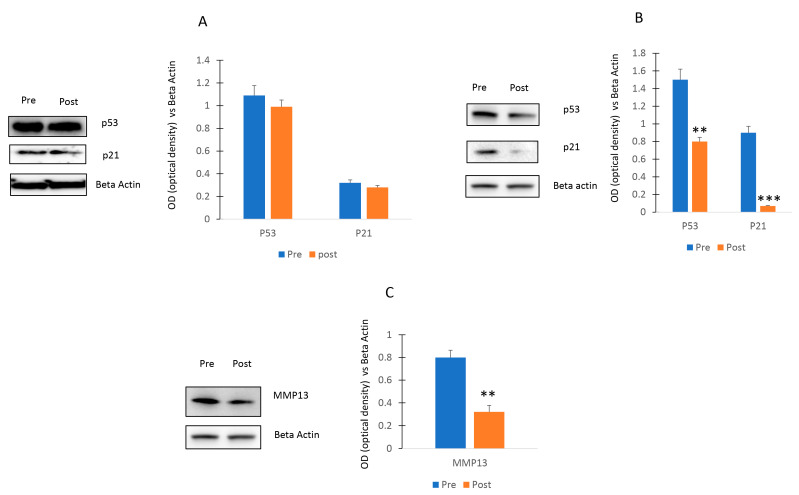
Effects of walking on p53/p21 axis. P53 and p21 levels were not affected in osteogenic cells (**A**) or decreased in chondrogenic cells (**B**) cultured Post sera. MMP13 levels (**C**) were reduced in chondroblastic progenitors stimulated with Post sera. The original blots are presented in Supplementary S1. Data are shown as mean ± standard deviation (SD); ** *p* < 0.01; *** *p* < 0.005.

As shown in Figure 7A, no differences of p53 and p21 levels were observed in osteogenic cells cultured with Pre or Post sera. In contrast, a decrease of the p53/p21 pathway (Figure 7B) and a reduction of MMP13 levels (Figure 7C) was observed in chondroblastic progenitors cultured with serum post exercise program.

## 3. Discussion

To assess the role of miR-146b in human progenitor cells, we analyzed its expression during commitment and differentiation of hMSCs. Our data showed that miR-146b is upregulated during adipogenic commitment compared to osteogenic or chondrogenic commitment. In addition, miR-146b is modulated during mesenchymal stem cells differentiation indicating that the modulation of miR-146b reflects processes related to differentiation. Interestingly, cell differentiation and aging have been associated with stress mechanisms [21,22,23,24,25]. In our study, we observed that forced expression of miR-146b increased Sestrin expression in MSCs, suggesting a potential link between miR-146b and stress conditions. A limitation of our study is the absence of experiments involving the inhibition of miR-146 to assess the modulation of Sestrin. Nevertheless, in the context of human THP-1 monocytic cells, research has indicated that suppressing miR-146b-5p interferes with the anti-inflammatory mechanisms facilitated by globular adiponectin [26]. Furthermore, studies have underscored the pivotal role of Sestrin induction in both cellular survival and the orchestration of anti-inflammatory reactions [27]. To evaluate the modulation of miR-146b during aging, another process associated with stress mechanisms, we used the zebrafish model. In recent years, the zebrafish model has become a valuable vertebrate model for the study of physiological and pathological mechanisms [28] in developmental biology and aging. An age-related decline in performance has been demonstrated using this in vivo model, with young (8–12 months), middle-aged (15–20 months), and old (25–30 months) zebrafish. Zebrafish is particularly useful in skeletal disease research due to its ease of gene manipulation, rapid development, low breeding cost, and its similarity in genetic and molecular mechanisms to humans [14,29,30,31]. Studies on fin development and regeneration in *Danio rerio* (zebrafish) have shown similarities to bone and cartilage differentiation observed in humans [32]. While mice and rats are commonly used experimental models to study bone alterations or repair, zebrafish has also emerged as a valuable model for studying skeletal alterations and pathophysiology [29,33,34,35,36,37]. 

Moreover, from an evolutionary point of view, miRNAs are highly conserved in vertebrate organisms, and using model organisms such as zebrafish provides insights into their role in various pathophysiological processes that can be translated into humans [38,39,40]. 

MicroRNAs have been demonstrated to induce post-transcriptional regulation of gene expression in bone development, regeneration, or alteration [41,42,43], including studies specifically focused on the role of microRNAs in zebrafish skeletal tissue [4,44,45,46,47]. The role of miR-146b has been investigated in several physio-pathological contexts, such as in angiogenesis, cancer, inflammation processes, and obesity disorders [48,49,50,51,52,53,54]. Recently, we reported that antioxidant treatment with methylsulfonylmethane reduces miR-146b expression in the caudal fin [14]. However, the miR-146b role during aging has not been extensively analyzed.

In this study, using the zebrafish model, we observed an increase in miR-146b expression during larval maturation. To evaluate miR-146b expression during aging in osteochondrogenic forming cells, we analyzed its expression in caudal fins of zebrafish, which consist of endochondral bony elements [55,56]. We observed a reduction in the expression of osteogenic and chondrogenic genes in the caudal fin, along with an increased expression of miR-146b in aged zebrafish, particularly associated with age-related phenotypes such as spine curvature. Furthermore, the increased expression of miR-146b was accompanied by increased expression of rank, a gene involved in osteoclast activation and bone resorption, in aged zebrafish. These results suggest that the modulation of miR-146b may reflect maturation and aging processes rather than differentiation itself. 

Circulating levels of miRNAs may mirror tissue molecules [57,58,59,60,61]. Therefore, in the current study, we examined the expression of miR-146b in human subjects. We observed reduced levels in males compared to females and higher circulating miR-146b levels in aged female. In males, however, this elevation became noticeable around the age of 42 and subsequently exhibited a relatively constant trend. This suggests that aging and gender may influence the presence of miR-146b into the bloodstream, possibly originating from different tissues. The modulation of miR-146b during aging has also been evaluated in cellular models such as macrophages or fibroblasts, yielding contrasting findings [62,63]. Specifically, it has been observed that the level of miR-146b decreases during aging in mouse macrophages, and this reduction contributes to inflammation. On the other hand, an increase in miR-146a/b has been reported in senescent fibroblasts that secrete elevated levels of inflammatory cytokines. In our cellular models, we observed increased levels of miR-146b during differentiation or with advancing age. Taken together, these data support that miR-146b levels reflect an inflammatory state associated with paraphysiological conditions occurring during the aging. Interestingly, when we evaluated Sestrin levels in MSCs stimulated with sera from female subjects, we observed reduced Sestrin levels in the presence of sera from elderly subjects. Accordingly, Zeng et al. found that Sestrin levels are downregulated with aging [64]. Thus, our findings suggest that the increased miR-146b expression observed in older subjects is an attempt by the cells to increase Sestrin levels. However, this upregulation of miR-146b does not induce an increase in Sestrins in older subjects.

Previous research has indicated that miR-146b plays a role in promoting osteoarthritis, a condition associated with aging, by inhibiting the alpha-2-macroglobulin expression [65]. Specifically, it has been observed that miR-146b reduces the expression of collagen II and aggrecan while increasing the expression of matrix metalloproteinase 3 (MMP-3) and MMP-13 enzymes in cartilage by targeting the chondroprotective molecule alpha-2-macroglobulin [65,66]. Additionally, studies have reported the downregulation of miR-146b during differentiation into chondrogenic lineage of skeletal stem cells while its levels are upregulated in osteoarthritis [67]. Interestingly, circulating miR-146a has been identified as potential biomarker of sarcopenia in older adults [68]. 

Furthermore, we demonstrated that a 4-week walking exercise program effectively modulates miR-146b levels as well as osteogenic and chondrogenic differentiation. Interestingly, these effects were observed even with low-intensity exercise such as walking. We observed a decrease in circulating miR-146b expression following the low-intensity exercise program. We also observed reduced Sestrin levels in MSCs in the presence of sera collected after the walking program. These data suggest that the walking program is not an activity that stimulates stressful conditions and therefore the demand for proteins associated with oxidative stress or inflammation, such as Sestrins, is reduced.

Population-based studies have reported that walking could be a protective factor against osteoarthritis in older [64] and middle-aged women [65]. It is widely acknowledged in literature that physical activity has a positive impact on reducing the progression of osteoarthritis [66] and preventing arthritis [67]. MMP-13 is considered the pivotal proteinase that marks osteoarthritis development and progression of osteoarthritis [69]. Cartilage damage is associated with degradation of the extracellular matrix due to altered levels of metalloproteinases, including MMP-13 [70]. Our study showed that a walking program in healthy women led to a reduction of MMP-13 expression in progenitors cells cultured with post-physical activity sera. 

Walking has several positive effects on functional parameters in patients with osteoarthritis and should be considered as a treatment for arthrosis [71].

Even low-intensity exercise has the potential to counteract aging, as we demonstrated modulation of a p53/p21 pathway in chondroblastic progenitors treated with post-exercise sera. These results can be explained by the immunomodulant and anti-aging effects of physical activity [72], which can directly influence signaling pathways associated with osteoarthritis [73].

Finally, it is widely recognized that women are more susceptible to osteoarthritis than men [74]. Therefore, future studies should focus on the investigating the role of miR-146b modulation and its differences between sexes to fill this gap in biomedical research [75].

## 4. Materials and Methods

### 4.1. Cells 

Human mesenchymal stem cells (hMSCs, PromoCell, Heidelberg, Germany), plated at a density of 5 × 10^4^ cells and cultured in the presence of mesenchymal stem cell growth medium (PromoCell, Heidelberg, Germany) or osteogenic or chondrogenic or adipogenic differentiation medium (PromoCell, Heidelberg, Germany), were incubated at 37 °C in a humidified atmosphere with 5% CO_2_ as previously reported [14,76]. Briefly, at 70% confluence, the specific culture medium was employed, namely Adipocyte StemPro*^®^* Basal Medium (Cat. Number A10410-01, ThermoFisher Scientific—New York 14072, NY, USA), Osteocyte/Chondrocyte StemPro*^®^* Basal Medium (Cat. Number A10069-01, ThermoFisher Scientific—New York, NY, USA), or MesenPro*^®^* Basal Medium (Cat.Number 12747-010, ThermoFisher—New York, NY, USA), depending on the type of differentiation. During adipogenic differentiation, the culture medium was enriched with an adipogenesis supplement (Cat. Number A10065-01, ThermoFisher Scientific—New York, NY, USA) and antibiotics (1% penicillin/streptomycin/Amphotericin B, Cat. Number 17-745E Lonza—Walkersville, MD, USA). In the case of osteogenic differentiation, the culture medium contained an osteogenesis supplement (Cat. Number A10066-01, ThermoFisher Scientific—New York, NY, USA), and antibiotics (1% penicillin/streptomycin/Amphotericin B, Cat. Number 17-745E Lonza—Walkersville, MD, USA). For chondrogenic differentiation, the culture medium included a chondrogenesis supplement (Cat-Number A10064-01, ThermoFisher Scientific—New York, NY, USA), and antibiotics (1% penicillin/streptomycin/Amphotericin B, Cat. Number 17-745E Lonza—Walkersville, MD, USA). Furthermore, for undifferentiated MSCs, MesenPro*^®^* Basal Medium was utilized, supplemented with MesenPro supplement (Cat. Number 12748-018, ThermoFisher Scientific—New York, NY, USA), 1% L-Glutamine (Cat. Number G7513, Merck—Darmstadt, Germany), and antibiotics (1% penicillin/streptomycin/Amphotericin B, Cat. Number 17-745E, Lonza, Walkersville, MD, USA).

We evaluated the effect of gain-function of miR-146b on the commitment or differentiation of MSCs by transfecting with a mirVana™ miR-146b mimic (Catalog number: 4464066 ID: MC25960, ThermoFisher Scientific—New York, NY, USA) or control/scramble. Transfection was carried at a cell confluency of 60–70% using Lipofectamine 3000 Reagent (L3000-008, Invitrogen by Thermo Fisher Scientific Baltics UAB, Vilnius, Lithuania). Then, 48 h post transfection, cells were collected and RNA was extracted as we previously performed [30].

To evaluate the effects of the walking program in MSCs differentiation, cells were cultured in presence of 5% serum collected from the walking program participants before (Pre) and after (Post) the activity.

### 4.2. Danio Rerio (Zebrafish) Model

The in vivo experiments were performed at the CIRSAL (Interdepartmental Center of Experimental Research Service—University of Verona, Verona, Italy). The procedures were performed under ethical authorization n. 662/2019-PR of 16/09/2019. The embryos were obtained from adults nacre zebrafish (ZFIN database ID: ZDB-ALT-990423-22) by performing standard procedures as previously reported [77,78]. The embryos were grown at 33 °C in water for 3 (30 embryos), 7 (30 embryos), and 14 (40 embryos) days post-fertilization (dpf). Then, the embryos were euthanized and collected in order to perform molecular analyses (30 embryos for each groups) or calcein staining (10 embryos at 14 dpf) as previously reported [79]. In addition, 2 months (*n* = 30) and 6 months (*n* = 30) zebrafish as well adult zebrafish ((young (10–12 months), middle-aged (20–24 months), and old (30–36 months)) were grown in water and, after the euthanasia, were collected for the molecular analyses (*n* = 30 for each group) and staining procedures (*n* = 10 each for young, middle-aged, and old groups) as previously reported [80].

### 4.3. RNA Extraction and Reverse Transcription

Pellets obtained from larvae or adults zebrafish were collected and stored at −80 °C as previously reported [79]. MiRNAs and RNA were extracted by using the miRNeasy Quiagen and RNAeasy Protect kits, respectively, as previously reported [30]. The RNA samples were analyzed to evaluate the concentration and purity by a Nanodrop instrument and reverse-transcribed into cDNA using Applied Biosystems Reverse Transcription kits (4368814 and 4366596, Thermo Fisher Scientific, Baltics UAB, Vilnius, Lithuania). Random hexamers (for RNA) or miRNA-specific primers (for miRNAs) were used according to the manufacturer’s protocol.

### 4.4. Real-Time PCR

Gene expression modulation was performed by real-time PCR analyses as previously reported [79]. The following primer sets were used: runx2a (fw GACGGTGGTGACGGTAATGG, rv TGCGGTGGGTTCGTGAATA, runx2b (fw CGGCTCCTACCAGTTCTCCA, rv CCATCTCCCTCCACTCCTCC), rank (fw GCACGGTTATTGTTGTTA, rv TATTCAGAGGTGGTGTTAT) (Invitrogen, Carlsbad, CA, USA). Predesigned, gene-specific primers sox9 Dr03112282_m1; miR-146b-5p, 474220; RNU44, 001094; Runx2_m1 (Hs01047973, 20X, FAM); Sox9-m1 (Hs00165814, 20X, FAM; sestrin1, hs00902782_m1; PPARG2, hs01115513_m1; β-actin (4326315E 20X VIC)) (Thermo Fisher Corporation, Waltham, MA, USA) were also used. Ct values for each reaction were determined using TaqMan SDS analysis software 7300 (Applied Biosystems; Foster City, CA, USA) as reported previously [14]. To calculate relative gene expression levels between different samples, we performed the analyses by using the 2^−ΔΔCT^ method as previously reported [14].

### 4.5. Western Blotting

The proteins were extracted by using Ripa buffer (Thermo Fisher Scientific, Waltham, MA, USA) and concentrations were calculated with BCA assay (Thermo Scientific, Waltham, MA, USA) as previously reported [76]. Protein samples were separated by sodium dodecyl sulfate–polyacrylamide gel electrophoresis (SDS PAGE) and then transferred onto polyvinylidene difluoride (PVDF) membranes (Thermo Fisher Scientific, Waltham, MA, USA). PVDF membranes were probed with the primary (β ACTIN (BA3R; Thermo Scientific, Waltham, MA, USA); sirtuin (PA5-23,063; Invitrogen, Waltham, MA, USA); SESN1 (PA5-98,142; Invitrogen, Waltham, MA, USA); SESN2 (ab-178518; Abcam, Cambridge, MA, USA); p53 (2524; Cell Signaling Technology, Danvers, MA, USA); p21 (M7202; Dako, Denmark A/S, Glostrup, Denmark); and secondary antibodies Anti-mouse (7076; Cell Signaling Technology) and Anti-rabbit (7074, Cell Signaling Technology). Signals were detected using chemiluminescence reagent (ECL, Millipore, Burlington, MA, USA). The images were captured with a LAS4000 Digital Image Scanning System (GE Healthcare, Little Chalfont, UK). Densitometric analyses were performed as we previously reported [76].

### 4.6. Zebrafish Staining

Calcein staining was performed at 14 days post fertilization as previously reported [79] and a Leica M205FA fluorescence microscope (Leica Microsystems, Wetzlar, Germany) camera was utilized to acquire and analyze the microscope images. Alizarin red to stain adults zebrafish was performed as previously described [79]. Briefly, euthanized fish were treated with fixative solution (5% formalin, 5% Triton X-100, 1% potassium hydroxide (KOH) and rocked for 48 h at room temperature (RT). The specimens were immersed in B-Staining medium (20% ethylene glycol and 1% KOH) and then in “B-Staining solution (0.05% Alizarin Red S, 20% ethylene glycol, 1% KOH) overnight at 20 °C. The specimens were then treated with clearing solution (20% Tween 20, 1% KOH) rocking for 12 h. The stocking was performed in glycerol 100%. 

### 4.7. Human Subjects

Samples from 12 women and 17 men without chronic medical conditions (median age: women 41.4 ± 7.2 (group a (*n* = 4) 27.8 ± 1.9; group b (*n* = 4) 41.2 ± 1.9; group c (*n* = 4) 52.8 ± 0.8); men 41.7 ± 9.7 (group a (*n* = 5) 28.4 ± 1.1; group b (*n* = 6) 42.5 ± 0.8; group c (*n* = 6) 52.8 ± 0.63) were analyzed to evaluate gender-dependent miR-146b-5p expression. Moreover, a cohort comprising 12 women engaged in moderate but consistent physical activity was meticulously chosen, and subsequent blood specimens were acquired. Specifically, samples were collected from women (median age 53.2 ± 6.6) who participated in a health-focused walking regimen supported by the Azienda Ospedaliera Universitaria Integrata of Verona. The blood samples were obtained both prior to and upon completion of the designated walking program. The subjects gave their written consent before the collection of blood samples, obtained by venipuncture. The study was approved by the local ethical committee of Azienda Ospedaliera Universitaria Integrata of Verona, Italy (number 1538; 3 December 2012). The study design and methods comply with the Declaration of Helsinki.

### 4.8. Walking Program and Physical Activity Assessment

The exercise program consisted of 3 walking sessions per week for a total of 4 weeks. Each session was supervised by a physiotherapist and comprised 10 min of low-intensity warm-up, 30 min of 6–8.5 km/h walking, and 5 min of cooling down. 

### 4.9. Serum Collection

In order to evaluate the modulation of circulating miR-146b-5p, sera were collected from 10 mL of fresh blood by centrifugation at 400× *g* as previously described [76]. Sera were frozen in aliquots at −80 °C until use. 

### 4.10. Circulating miRNAs 

Circulating miRNAs were extracted from collected sera by using the miRNeasy Serum/Plasma Advanced Kit (Qiagen, Hilden, Germany) as previously reported [77]. miRNAs samples were quantified by using the “Qubit™ RNA HS assay kit” (Invitrogen, Carlsbad, CA, USA). The extracted miRNAs were reverse transcribed using the TaqMan microRNA Reverse Transcription kit (Thermofisher Corporation, Waltham, MA, USA) as previously reported ([77]). The samples were stored at −80 °C until real-time PCR analyses as described above.

### 4.11. Oil Red O Staining

Positive adipogenic cells were evaluated by using Oil Red O (ORO) staining according to the manufacturer’s instructions. The total area of red pixels in cell was determined by using the IMAGE J (Java2HTML Version 1.5) image analysis as previously reported [76]. In particular, three different fields/slides at magnification of 40× for each condition were evaluated. The red pixel-stained area was calculated by the IMAGE J (Java2HTML Version 1.5) analyses and it was expressed as percentage respect to total area. 

### 4.12. Three-Dimensional (3D) Cultures

In addition, 3D cultures were performed by using scaffolds (VITVO^®^_Rigenerand) to analyze the interaction between MSCs in osteogenic differentiation and MSCs in chondrogenic differentiation under blood serum stimulation. Before inserting the cells into the VITVO^®^, MSCs were cultured in flasks with osteogenic or chondrogenic differentiation medium for 3 days. On the fourth day, the cells were stained with vital fluorescent dyes (Vybrant Cell Labeling Solution_Invitrogen): MSCs in osteogenic differentiation were stained with DiI (emission 565 nm, red) whereas MSCs in chondrogenic differentiation were stained with DiO (emission 501 nm, green). According to the manufacture’s indication, the VITVO^®^ were initially primed, filling them with basal culture medium, ensuring the complete wetting of the 3D matrix. Proceeding with the VITVO^®^ 3D Bioreactor protocol, an equivalent number of osteogenic and chondrogenic differentiated MSCs, was injected in the VITVO^®^. The scaffold VITVO^®^ was cultured in presence of 5% serum PRE or 5% serum POST. The two bioreactors were incubated at 37 °C at 5% of CO_2_ for 48 h. 

The scaffolds were observed through the EVOS Fluorescence Microscope (Life Technologies, ThermoFisher Scientific—New York, NY, USA). A picture of the same area was taken through GFP filter to see chondrogenic differentiated MSCs and through RFP filter to see the osteogenic differentiated MSCs. Merged pictures, with the two filters both activated, were taken. 

The number of red (osteogenic) or green (chondrogenic) cells was evaluated in six randomly selected squares of 100^2^ microns by using Image J (Java2HTML Version 1.5) analysis. 

### 4.13. Statistical Analysis

Data were expressed reporting mean ± SD. Statistical analysis was performed by using Student’s paired *t*-test comparing sample to the control. Differences were considered statistically significant for values of *p* < 0.05. The analyses were carried out at least three times. To analyze the data, SPSS for Windows, version 22.0 (SPSS Inc., Chicago, IL, USA) was used.

## 5. Conclusions

In conclusion, our study highlights the potential of miR-146b-5p as an age-related marker and its relevance in identifying strategies, such as physical activity, that can counteract age-related degenerative processes. Further research is warranted to elucidate the underlying mechanisms and explore the therapeutic potential of miR-146b-5p modulation in the context of aging and degenerative diseases.

## Data Availability

All data generated or analyzed during this study are included in this published article.

## Supplementary Materials

The following supporting information can be downloaded at: https://www.mdpi.com/article/10.3390/ijms241713163/s1.

## Abbreviations

hMSC	Human mesenchymal stem cells
dpf	Days post-fertilization
SESN	Sestrin
KOH	Potassium hydroxide
ARS	Alizarin red staining
ORO	Oil Red O staining
GFP	Green-excited fluorophores
RFP	Red-excited fluorophores
Rank	Receptor for RANK-Ligand

## References

[1] Heid J., Cencioni C., Ripa R., Baumgart M., Atlante S., Milano G., Scopece A., Kuenne C., Guenther S., Azzimato V. (2017). Age-dependent increase of oxidative stress regulates microRNA-29 family preserving cardiac health. Sci. Rep..

[2] Campisi J., Kapahi P., Lithgow G.J., Melov S., Newman J.C., Verdin E. (2019). From discoveries in ageing research to therapeutics for healthy ageing. Nature.

[3] Montesanto A., Dato S., Bellizzi D., Rose G., Passarino G. (2012). Epidemiological, genetic and epigenetic aspects of the research on healthy ageing and longevity. Immun. Ageing.

[4] Shi L., Su Y., Zheng Z., Qi J., Wang W., Wang C. (2022). miR-146b-5p promotes colorectal cancer progression by targeting TRAF6. Exp. Ther. Med..

[5] De Santa-Inez D.C., Fuziwara C.S., Saito K.C., Kimura E.T. (2021). Targeting the highly expressed microRNA miR-146b with CRISPR/Cas9n gene editing system in thyroid cancer. Int. J. Mol. Sci..

[6] Geraldo M.V., Yamashita A.S., Kimura E.T. (2012). MicroRNA miR-146b-5p regulates signal transduction of TGF-β by repressing SMAD4 in thyroid cancer. Oncogene.

[7] Chen Z., Wang K., Huang J., Zheng G., Lv Y., Luo N., Liang M., Huang L. (2018). Upregulated serum MiR-146b serves as a biomarker for acute ischemic stroke. Cell. Physiol. Biochem..

[8] Chen L., Dai Y.-M., Ji C.-B., Yang L., Shi C.-M., Xu G.-F., Pang L.-X., Huang F.-Y., Zhang C.-M., Guo X.-R. (2014). MiR-146b is a regulator of human visceral preadipocyte proliferation and differentiation and its expression is altered in human obesity. Mol. Cell. Endocrinol..

[9] Lee H.-M., Kim T.S., Jo E.-K. (2016). MiR-146 and miR-125 in the regulation of innate immunity and inflammation. BMB Rep..

[10] Paterson M.R., Geurts A.M., Kriegel A.J. (2019). miR-146b-5p has a sex-specific role in renal and cardiac pathology in a rat model of chronic kidney disease. Kidney Int..

[11] Kishi S., Slack B.E., Uchiyama J., Zhdanova I.V. (2009). Zebrafish as a genetic model in biological and behavioral gerontology: Where development meets aging in vertebrates—A mini-review. Gerontology.

[12] Gerhard G.S., Kauffman E.J., Wang X., Stewart R., Moore J.L., Kasales C.J., Demidenko E., Cheng K.C. (2002). Life spans and senescent phenotypes in two strains of Zebrafish (Danio rerio). Exp. Gerontol..

[13] Bhattacharya M., Sharma A.R., Sharma G., Patra B.C., Nam J.-S., Chakraborty C., Lee S.-S. (2017). The crucial role and regulations of miRNAs in zebrafish development. Protoplasma.

[14] Dalle Carbonare L., Bertacco J., Marchetto G., Cheri S., Deiana M., Minoia A., Tiso N., Mottes M., Valenti M.T. (2021). Methylsulfonylmethane enhances MSC chondrogenic commitment and promotes pre-osteoblasts formation. Stem Cell Res. Ther..

[15] Uemoto T., Abe G., Tamura K. (2020). Regrowth of zebrafish caudal fin regeneration is determined by the amputated length. Sci. Rep..

[16] Turishcheva E., Vildanova M., Onishchenko G., Smirnova E. (2022). The Role of Endoplasmic Reticulum Stress in Differentiation of Cells of Mesenchymal Origin. Biochemistry.

[17] Livshits G., Kalinkovich A. (2023). A cross-talk between sestrins, chronic inflammation and cellular senescence governs the development of age-associated sarcopenia and obesity. Ageing Res. Rev..

[18] Azevedo A.S., Grotek B., Jacinto A., Weidinger G., Saúde L. (2011). The regenerative capacity of the zebrafish caudal fin is not affected by repeated amputations. PLoS ONE.

[19] Chia C.W., Sherman-Baust C.A., Larson S.A., Pandey R., Withers R., Karikkineth A.C., Zukley L.M., Campisi J., Egan J.M., Sen R. (2021). Age-associated expression of p21and p53 during human wound healing. Aging Cell.

[20] Hu Q., Ecker M. (2021). Overview of MMP-13 as a Promising Target for the Treatment of Osteoarthritis. Int. J. Mol. Sci..

[21] Kudryavtseva A.V., Krasnov G.S., Dmitriev A.A., Alekseev B.Y., Kardymon O.L., Sadritdinova A.F., Fedorova M.S., Pokrovsky A.V., Melnikova N.V., Kaprin A.D. (2016). Mitochondrial dysfunction and oxidative stress in aging and cancer. Oncotarget.

[22] Hajam Y.A., Rani R., Ganie S.Y., Sheikh T.A., Javaid D., Qadri S.S., Pramodh S., Alsulimani A., Alkhanani M.F., Harakeh S. (2022). Oxidative Stress in Human Pathology and Aging: Molecular Mechanisms and Perspectives. Cells.

[23] Arrigo A.P. (2005). In search of the molecular mechanism by which small stress proteins counteract apoptosis during cellular differentiation. J. Cell. Biochem..

[24] Mody N., Parhami F., Sarafian T.A., Demer L.L. (2001). Oxidative stress modulates osteoblastic differentiation of vascular and bone cells. Free. Radic. Biol. Med..

[25] Lee J., Cho Y.S., Jung H., Choi I. (2018). Pharmacological regulation of oxidative stress in stem cells. Oxidative Med. Cell. Longev..

[26] Hulsmans M., Van Dooren E., Mathieu C., Holvoet P. (2012). Decrease of miR-146b-5p in monocytes during obesity is associated with loss of the anti-inflammatory but not insulin signaling action of adiponectin. PLoS ONE.

[27] Lee S., Pham D.-V., Park P.-H. (2022). Sestrin2 induction contributes to anti-inflammatory responses and cell survival by globular adiponectin in macrophages. Arch. Pharmacal Res..

[28] Tonelli F., Bek J.W., Besio R., De Clercq A., Leoni L., Salmon P., Coucke P.J., Willaert A., Forlino A. (2020). Zebrafish: A resourceful vertebrate model to investigate skeletal disorders. Front. Endocrinol..

[29] Valenti M.T., Marchetto G., Mottes M., Carbonare L.D. (2020). Zebrafish: A suitable tool for the study of cell signaling in bone. Cells.

[30] Dalle Carbonare L., Bertacco J., Minoia A., Cominacini M., Bhandary L., Elia R., Gambaro G., Mottes M., Valenti M.T. (2022). Modulation of mir-204 expression during chondrogenesis. Int. J. Mol. Sci..

[31] Kwon R.Y., Watson C.J., Karasik D. (2019). Using zebrafish to study skeletal genomics. Bone.

[32] Marí-Beffa M., Santamaría J.A., Murciano C., Santos-Ruiz L., Andrades J.A., Guerado E., Becerra J. (2007). Zebrafish fins as a model system for skeletal human studies. Sci. World J..

[33] Dietrich K., Fiedler I.A., Kurzyukova A., López-Delgado A.C., McGowan L.M., Geurtzen K., Hammond C.L., Busse B., Knopf F. (2021). Skeletal biology and disease modeling in zebrafish. J. Bone Miner. Res..

[34] Moss J.J., Hammond C.L., Lane J.D. (2020). Zebrafish as a model to study autophagy and its role in skeletal development and disease. Histochem. Cell Biol..

[35] Choi T.-Y., Choi T.-I., Lee Y.-R., Choe S.-K., Kim C.-H. (2021). Zebrafish as an animal model for biomedical research. Exp. Mol. Med..

[36] Bergen D.J.M., Kague E., Hammond C.L. (2019). Zebrafish as an emerging model for osteoporosis: A primary testing platform for screening new osteo-active compounds. Front. Endocrinol..

[37] Sojan J.M., Gundappa M.K., Carletti A., Gaspar V., Gavaia P., Maradonna F., Carnevali O. (2022). Zebrafish as a Model to Unveil the Pro-Osteogenic Effects of Boron-Vitamin D3 Synergism. Front. Nutr..

[38] Nakamura Y., He X., Kato H., Wakitani S., Kobayashi T., Watanabe S., Iida A., Tahara H., Warman M.L., Watanapokasin R. (2012). Sox9 is upstream of microRNA-140 in cartilage. Appl. Biochem. Biotechnol..

[39] Kim K.M., Park S.J., Jung S.H., Kim E.J., Jogeswar G., Ajita J., Rhee Y., Kim C.H., Lim S.K. (2012). miR-182 is a negative regulator of osteoblast proliferation, differentiation, and skeletogenesis through targeting FoxO1. J. Bone Miner. Res..

[40] Lawrence E.A., Hammond C.L., Blain E.J. (2020). Potential of zebrafish as a model to characterise MicroRNA profiles in mechanically mediated joint degeneration. Histochem. Cell Biol..

[41] Lian J.B., Stein G.S., van Wijnen A.J., Stein J.L., Hassan M.Q., Gaur T., Zhang Y. (2012). MicroRNA control of bone formation and homeostasis. Nat. Rev. Endocrinol..

[42] Kapinas K., Delany A.M. (2011). MicroRNA biogenesis and regulation of bone remodeling. Thromb. Haemost..

[43] Cheng V.K.-F., Au P.C.-M., Tan K.C., Cheung C.-L. (2019). MicroRNA and human bone health. JBMR Plus.

[44] Ju L., Zhou Z., Jiang B., Lou Y., Zhang Z. (2017). miR-21 is involved in skeletal deficiencies of zebrafish embryos exposed to polychlorinated biphenyls. Environ. Sci. Pollut. Res..

[45] Gan S., Huang Z., Liu N., Su R., Xie G., Zhong B., Zhang K., Wang S., Hu X., Zhang J. (2016). MicroRNA-140-5p impairs zebrafish embryonic bone development via targeting BMP-2. FEBS Lett..

[46] Papaioannou G., Inloes J.B., Nakamura Y., Paltrinieri E., Kobayashi T. (2013). let-7 and miR-140 microRNAs coordinately regulate skeletal development. Proc. Natl. Acad. Sci. USA.

[47] Sera S.R., Zur Nieden N.I. (2017). microRNA regulation of skeletal development. Curr. Osteoporos. Rep..

[48] Shen H., Wang D., Zhan M., Ding H., Zhao H. (2022). MicroRNA-146a and microRNA-146b deficiency correlates with exacerbated disease activity, and their longitude increment relates to etanercept response in psoriasis patients. J. Clin. Lab. Anal..

[49] Tekcan E., Kara N., Aydın H.M., Abur Ü., Abbaszadeh M. (2022). Evaluation of the promoter methylation status of hypoxia factor 3A and interleukin-6 genes and expression levels of mir-130b and mir-146b in childhood obesity. Rev. Assoc. Méd. Bras..

[50] Fullerton J.L., Cosgrove C.C., Rooney R.A., Work L.M. (2022). Extracellular vesicles and their microRNA cargo in ischaemic stroke. J. Physiol..

[51] Soler-Botija C., Monguió-Tortajada M., Munizaga-Larroudé M., Gálvez-Montón C., Bayes-Genis A., Roura S. (2022). Mechanisms governing the therapeutic effect of mesenchymal stromal cell-derived extracellular vesicles: A scoping review of preclinical evidence. Biomed. Pharmacother..

[52] Katakowski M., Zheng X., Jiang F., Rogers T., Szalad A., Chopp M. (2010). MiR-146b-5p suppresses EGFR expression and reduces In Vitro migration and invasion of glioma. Cancer Investig..

[53] Li Y., Zhang H., Dong Y., Fan Y., Li Y., Zhao C., Wang C., Liu J., Li X., Dong M. (2017). MiR-146b-5p functions as a suppressor miRNA and prognosis predictor in non-small cell lung cancer. J. Cancer.

[54] Wang H., Jiang M., Xu Z., Huang H., Gong P., Zhu H., Ruan C. (2015). miR-146b-5p promotes VSMC proliferation and migration. Int. J. Clin. Exp. Pathol..

[55] Ando K., Shibata E., Hans S., Brand M., Kawakami A. (2017). Osteoblast production by reserved progenitor cells in zebrafish bone regeneration and maintenance. Dev. Cell.

[56] Sehring I.M., Weidinger G. (2020). Recent advancements in understanding fin regeneration in zebrafish. Wiley Interdiscip. Rev. Dev. Biol..

[57] Loosen S.H., Schueller F., Trautwein C., Roy S., Roderburg C. (2017). Role of circulating microRNAs in liver diseases. World J. Hepatol..

[58] Van Empel V.P., De Windt L.J., Martins P.A. (2012). Circulating miRNAs: Reflecting or affecting cardiovascular disease?. Curr. Hypertens. Rep..

[59] Anfossi S., Babayan A., Pantel K., Calin G.A. (2018). Clinical utility of circulating non-coding RNAs—An update. Nat. Rev. Clin. Oncol..

[60] Ward J., Kanchagar C., Veksler-Lublinsky I., Lee R.C., McGill M.R., Jaeschke H., Curry S.C., Ambros V.R. (2014). Circulating microRNA profiles in human patients with acetaminophen hepatotoxicity or ischemic hepatitis. Proc. Natl. Acad. Sci. USA.

[61] Basati G., Razavi A.E., Pakzad I., Malayeri F.A. (2016). Circulating levels of the miRNAs, miR-194, and miR-29b, as clinically useful biomarkers for colorectal cancer. Tumor Biol..

[62] Bhaumik D., Scott G.K., Schokrpur S., Patil C.K., Orjalo A.V., Rodier F., Lithgow G.J., Campisi J. (2009). MicroRNAs miR-146a/b negatively modulate the senescence-associated inflammatory mediators IL-6 and IL-8. Aging.

[63] Santeford A., Lee A.Y., Sene A., Hassman L.M., Sergushichev A.A., Loginicheva E., Artyomov M.N., Ruzycki P.A., Apte R.S. (2021). Loss of Mir146b with aging contributes to inflammation and mitochondrial dysfunction in thioglycollate-elicited peritoneal macrophages. eLife.

[64] Zeng N., D’Souza R.F., Mitchell C.J., Cameron-Smith D. (2018). Sestrins are differentially expressed with age in the skeletal muscle of men: A cross-sectional analysis. Exp. Gerontol..

[65] Liu X., Liu L., Zhang H., Shao Y., Chen Z., Feng X., Fang H., Zhao C., Pan J., Zhang H. (2019). MiR-146b accelerates osteoarthritis progression by targeting alpha-2-macroglobulin. Aging.

[66] Zhang Y., Wei X., Browning S., Scuderi G., Hanna L.S., Wei L. (2017). Targeted designed variants of alpha-2-macroglobulin (A2M) attenuate cartilage degeneration in a rat model of osteoarthritis induced by anterior cruciate ligament transection. Arthritis Res. Ther..

[67] Budd E., de Andrés M.C., Sanchez-Elsner T., Oreffo R.O.C. (2017). MiR-146b is down-regulated during the chondrogenic differentiation of human bone marrow derived skeletal stem cells and up-regulated in osteoarthritis. Sci. Rep..

[68] Liu H.-C., Han D.-S., Hsu C.-C., Wang J.-S. (2021). Circulating MicroRNA-486 and MicroRNA-146a serve as potential biomarkers of sarcopenia in the older adults. BMC Geriatr..

[69] Goldring M.B. (2012). Chondrogenesis, chondrocyte differentiation, and articular cartilage metabolism in health and osteoarthritis. Ther. Adv. Musculoskelet. Dis..

[70] Kong H., Wang X.-Q., Zhang X.-A. (2022). Exercise for osteoarthritis: A literature review of pathology and mechanism. Front. Aging Neurosci..

[71] Hiyama Y., Yamada M., Kitagawa A., Tei N., Okada S. (2012). A four-week walking exercise programme in patients with knee osteoarthritis improves the ability of dual-task performance: A randomized controlled trial. Clin. Rehabil..

[72] Englund D.A., Sakamoto A.E., Fritsche C.M., Heeren A.A., Zhang X., Kotajarvi B.R., Lecy D.R., Yousefzadeh M.J., Schafer M.J., White T.A. (2021). Exercise reduces circulating biomarkers of cellular senescence in humans. Aging Cell.

[73] Pedraza-Vázquez G., Mena-Montes B., Hernández-Álvarez D., Gómez-Verjan J.C., Toledo-Pérez R., López-Teros M.T., Königsberg M., Gómez-Quiroz L.E., Luna-López A. (2023). A low-intensity lifelong exercise routine changes miRNA expression in aging and prevents osteosarcopenic obesity by modulating inflammation. Arch. Gerontol. Geriatr..

[74] Cui A., Li H., Wang D., Zhong J., Chen Y., Lu H. (2020). Global, regional prevalence, incidence and risk factors of knee osteoarthritis in population-based studies. eClinicalMedicine.

[75] Tschon M., Contartese D., Pagani S., Borsari V., Fini M. (2021). Gender and sex are key determinants in osteoarthritis not only confounding variables. A systematic review of clinical data. J. Clin. Med..

[76] Carbonare L.D., Mottes M., Cheri S., Deiana M., Zamboni F., Gabbiani D., Schena F., Salvagno G.L., Lippi G., Valenti M.T. (2019). Increased Gene Expression of RUNX2 and SOX9 in Mesenchymal Circulating Progenitors Is Associated with Autophagy during Physical Activity. Oxidative Med. Cell. Longev..

[77] Carbonare L.D., Dorelli G., Vigni V.L., Minoia A., Bertacco J., Cheri S., Deiana M., Innamorati G., Cominacini M., Tarperi C. (2022). Physical Activity Modulates miRNAs Levels and Enhances MYOD Expression in Myoblasts. Stem Cell Rev. Rep..

[78] Whitlock K.E., Westerfield M. (2000). The olfactory placodes of the zebrafish form by convergence of cellular fields at the edge of the neural plate. Development.

[79] Carbonare L.D., Bertacco J., Gaglio S.C., Minoia A., Cominacini M., Cheri S., Deiana M., Marchetto G., Bisognin A., Gandini A. (2022). Fisetin: An Integrated Approach to Identify a Strategy Promoting Osteogenesis. Front. Pharmacol..

[80] Choi S.-W., Son Y.-J., Yun J.-M., Kim S.H. (2012). Fisetin Inhibits Osteoclast Differentiation via Downregulation of p38 and c-Fos-NFATc1 Signaling Pathways. Evid.-Based Complement. Altern. Med..

