# Supplementary material for: Modulation of miR-146b Expression during Aging and the Impact of Physical Activity on Its Expression and Chondrogenic Progenitors
Source: Int J Mol Sci. 2023 Aug 24;24(17):13163. doi: 10.3390/ijms241713163 (PMC10488278; doi:10.3390/ijms241713163)
Supplement: Supplementary file 1 [file ijms-24-13163-s001.zip › ijms-2567901-supplementary.pdf]

Thermo fisher

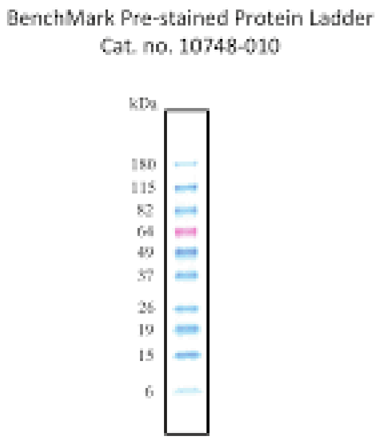

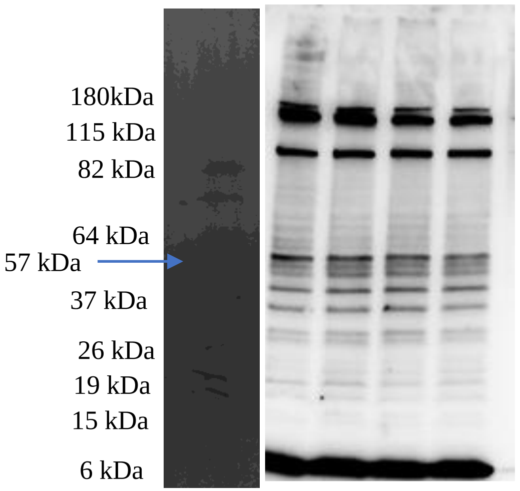

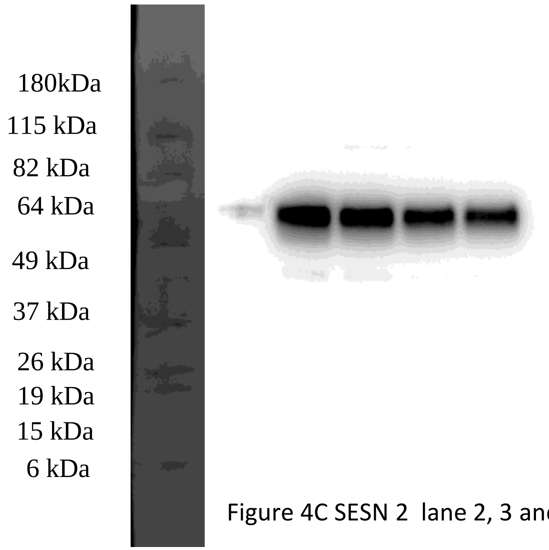

Figure 4C SESN 2 lane 2, 3 and 4

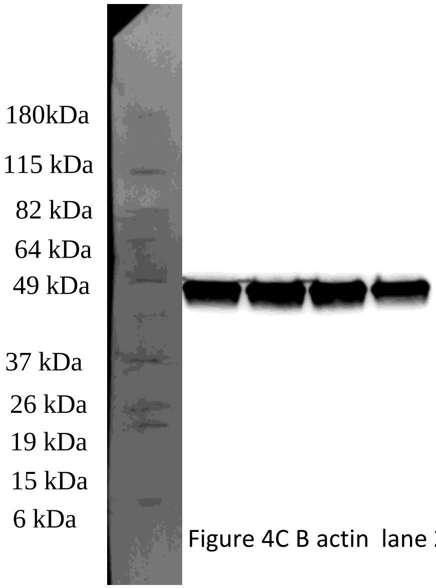

Figure 4C B actin lane 2, 3 and 4

Figure 4C SESN 1 (57kd) lane 2, 3 and 4

NeoPRO 10 Prestained Protein Ladder NB-59-0002

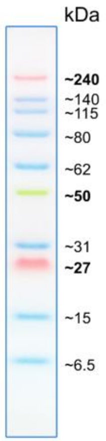

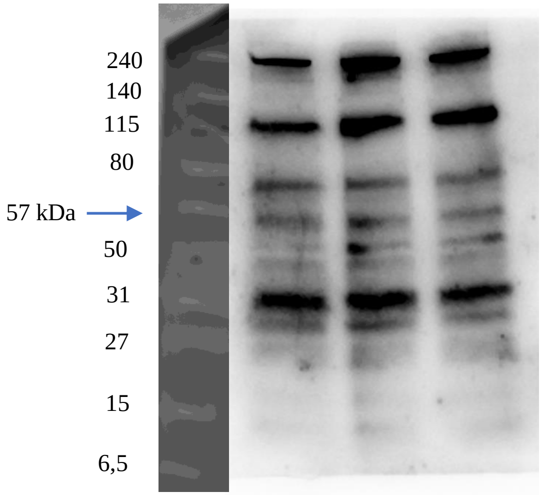

Figure 5B SESN1 lane 2 and 3

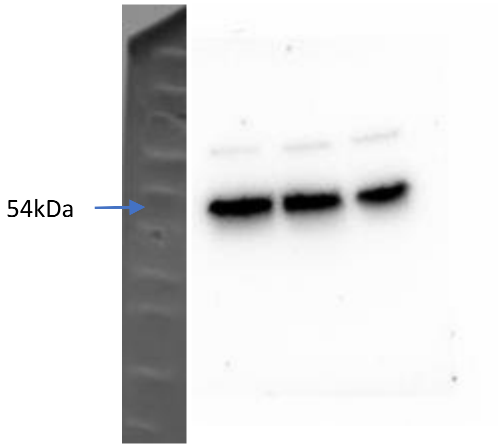

Figure 5B SESN2 lane 2 and 3

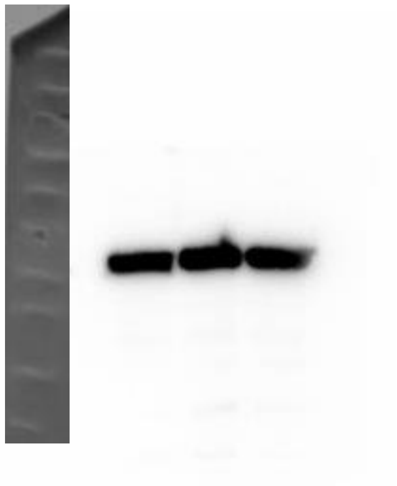

Figure 5B actin lane 1 and 2

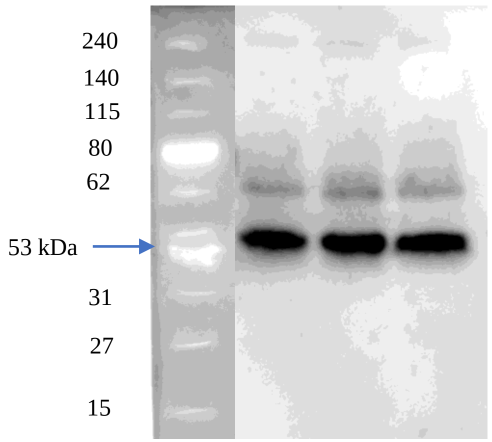

Figure 7A P53 lane 2 and 3

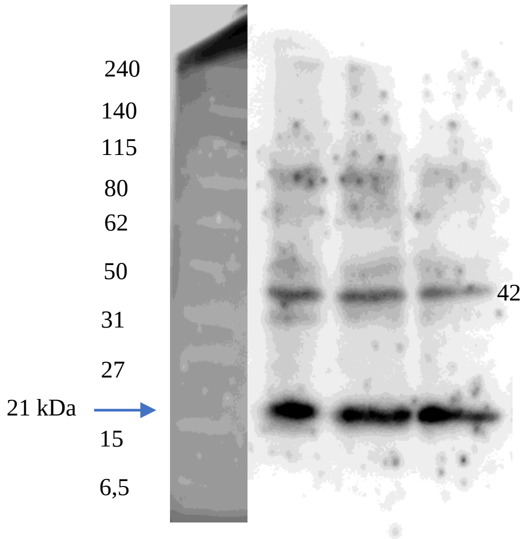

Figure 7A p21 lane 2 and 3

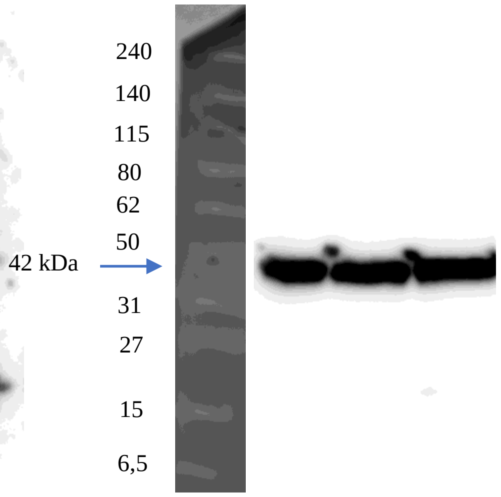

Figure 7A B actin lane 2 and 3

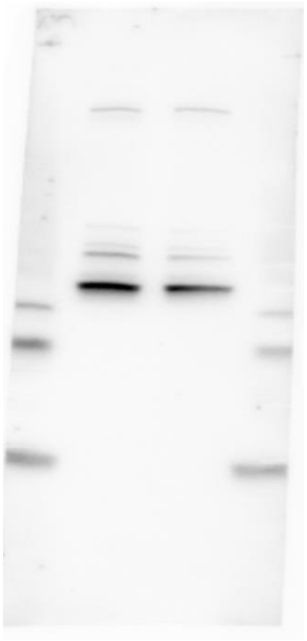

Figure 7B P53 lane 2 and 3

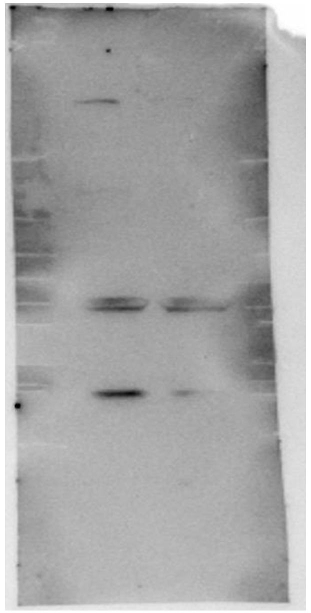

Figure 7B p21 lane 2 and 3

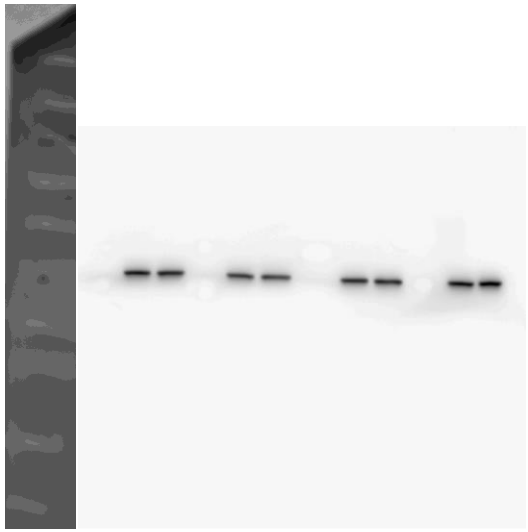

Figure 7C B actin lane 4 and 5

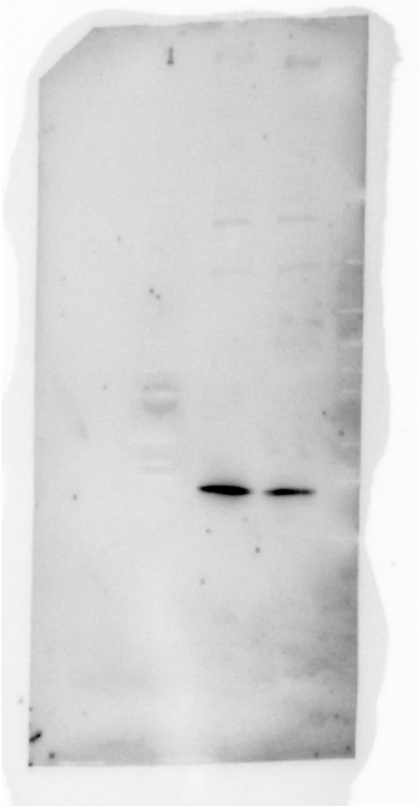

Figure 7C MMP13 lane 2 and 3

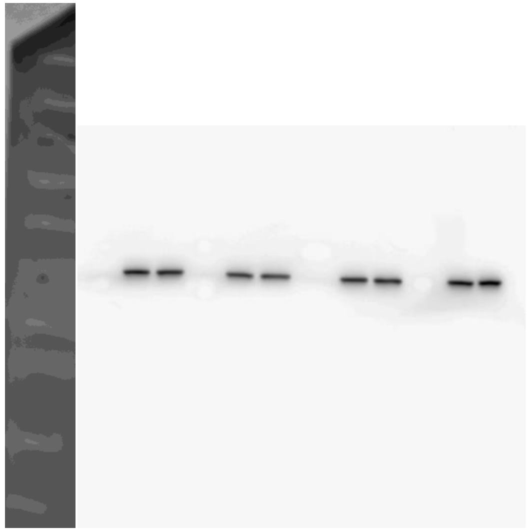

Figure 7C B actin lane 2 and 3
